# Supplementary material for: Molecular basis for RNA polymerase-dependent transcription complex recycling by the helicase-like motor protein HelD
Source: Nat Commun. 2020 Dec 18;11:6420. doi: 10.1038/s41467-020-20157-5 (PMC7749167; doi:10.1038/s41467-020-20157-5)
Supplement: Supplementary file 1 — Supplementary Information [file 41467_2020_20157_MOESM1_ESM.pdf]

SUPPLEMENTARY INFORMATION

Molecular Basis for RNA Polymerase-Dependent Transcription Complex Recycling By The Helicase-like Motor Protein HelD

Timothy Newing[1], Aaron J. Oakley[1], Michael Miller[2], Catherine Dawson[2], Simon H.J. Brown[1], James C. Bouwer[1], Gökhan Tolun[1]*, Peter J. Lewis[2]*

[1]Molecular Horizons and School of Chemistry and Molecular Bioscience, University of Wollongong, and Illawarra Health and Medical Research Institute, Wollongong, NSW 2522, Australia

[2]School of Environmental and Life Sciences, University of Newcastle, Callaghan, NSW 2308, Australia

*For correspondence: Peter.Lewis@newcastle.edu.au or gokhan_tolun@uow.edu.au

Supplementary Figure 1.

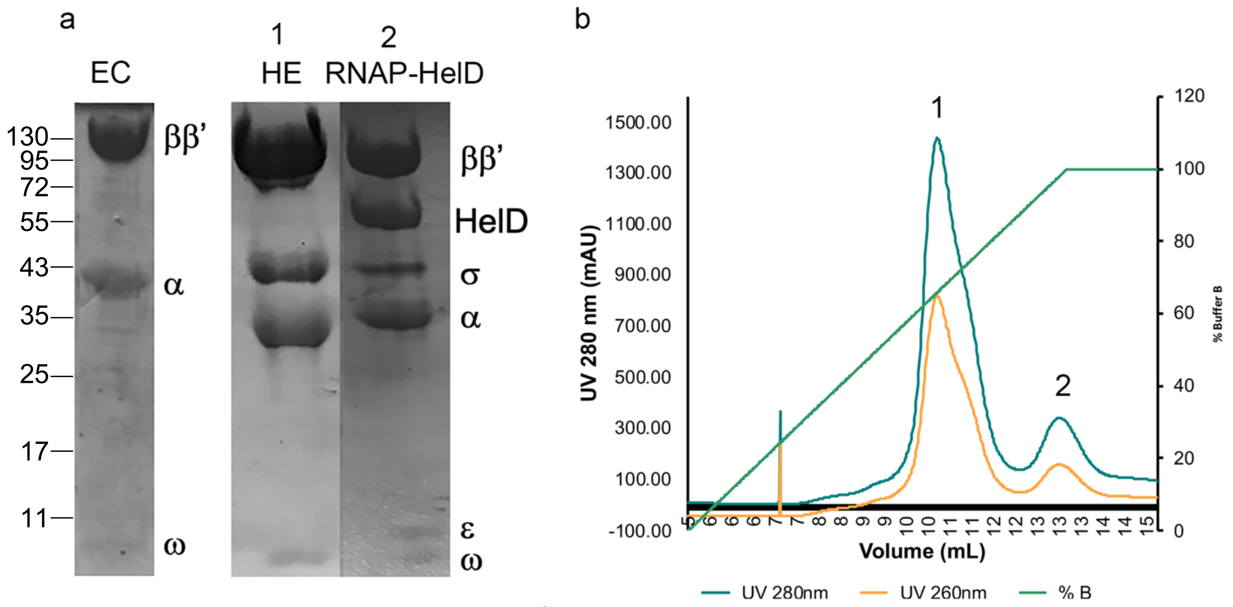

Supplementary Figure 1. Purification of *B. subtilis* RNAP core and RNAP-HelD complexes. a, *B. subtilis* RNAP core (EC), holoenzyme ($\alpha_2\beta\beta\omega\sigma^A$; HE) and RNAP-HelD complexes were purified as described in Methods and an image of Coomassie blue stained SDS-PAGE samples is shown. Molecular weight (kDa) is indicated on the left of the EC lane. Source Data are provided as Source Data file Uncropped Gels. **b,** The final step of the purification of the RNAP-HelD complex was performed by heparin sepharose chromatography which separated holoenzyme (HE; peak 1) from HelD complex (peak 2). The HE complex contained no detectable ε and the RNAP-HelD complex contained sub-stoichiometric levels of σ^A which was not detectable in cryoEM reconstructions.

Supplementary Figure 2.

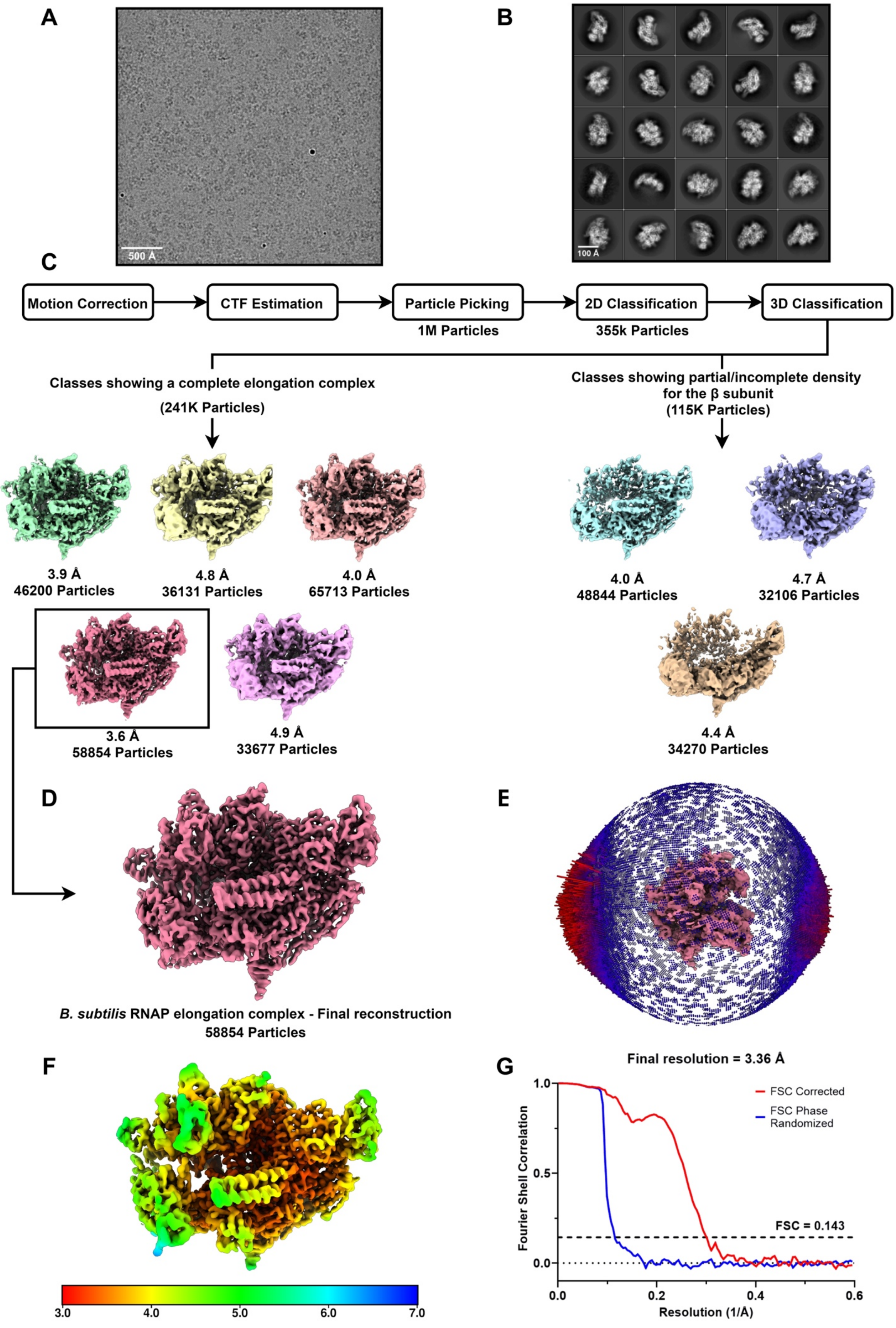

Supplementary Figure 2: Reconstruction of the *B. subtilis* RNAP elongation complex.
a, Representative micrograph **b**, Representative class averages, ordered by particle number (descending, left to right, top to bottom). Scale bars (Å) are shown in the bottom left corner of panels **a** and **b**. **c**, Simplified workflow for preprocessing and classification of the RNAP elongation complex. **d**, Final refined, unsharpened map of the *B. subtilis* RNAP elongation complex. Final reconstruction was performed with a subset of 58,854 particles. **e**, Orientational distribution for the RNAP elongation complex. Relative particle frequency per orientation is shown by the height and color of the cylinder (higher frequency = longer cylinder, red, lower frequency = shorter cylinder, blue). **f**, Local resolution across the final RNAP elongation complex reconstruction. Local resolution estimation was performed in RELION. **g**, Fourier shell correlation curves for the RNAP elongation complex reconstruction. Resolution estimation was performed in RELION using the GS-FSC criterion.

Supplementary Table 1.
Quantitation of DNA and Protein in EC and RNAP-HelD complexes. Quantitation was determined using a Qubit fluorometer with the protein and HS DNA assay procedures. Raw data, averages and standard deviations are on the left side of the table, with relative DNA:Protein and DNA content in the EC *vs* RNAP-HelD complex on the right side.

**Sample**	**DNA (ng/mL)***	**Protein (ng/mL)**
EC 1	9080	3000000
EC 2	11300	3840000
EC 3	11060	2640000
**EC Ave**	**10480**	**3160000**
St Dev	1218	615792
RNAP-HelD 1	216	1350000
RNAP-HelD 2	326	1460000
RNAPHelD 3	316	1180000
**RNAP-HelD Ave**	**286**	**1330000**
St Dev	61	141067

**Sample**	**DNA:Protein (ng/ng)**
EC	0.00332
RNAP-HelD	0.00022
EC:RNAP-HelD	15.4

* DNA content is likely an under-estimation due to the small amount of duplex present in the EC.

Supplementary Figure 3.

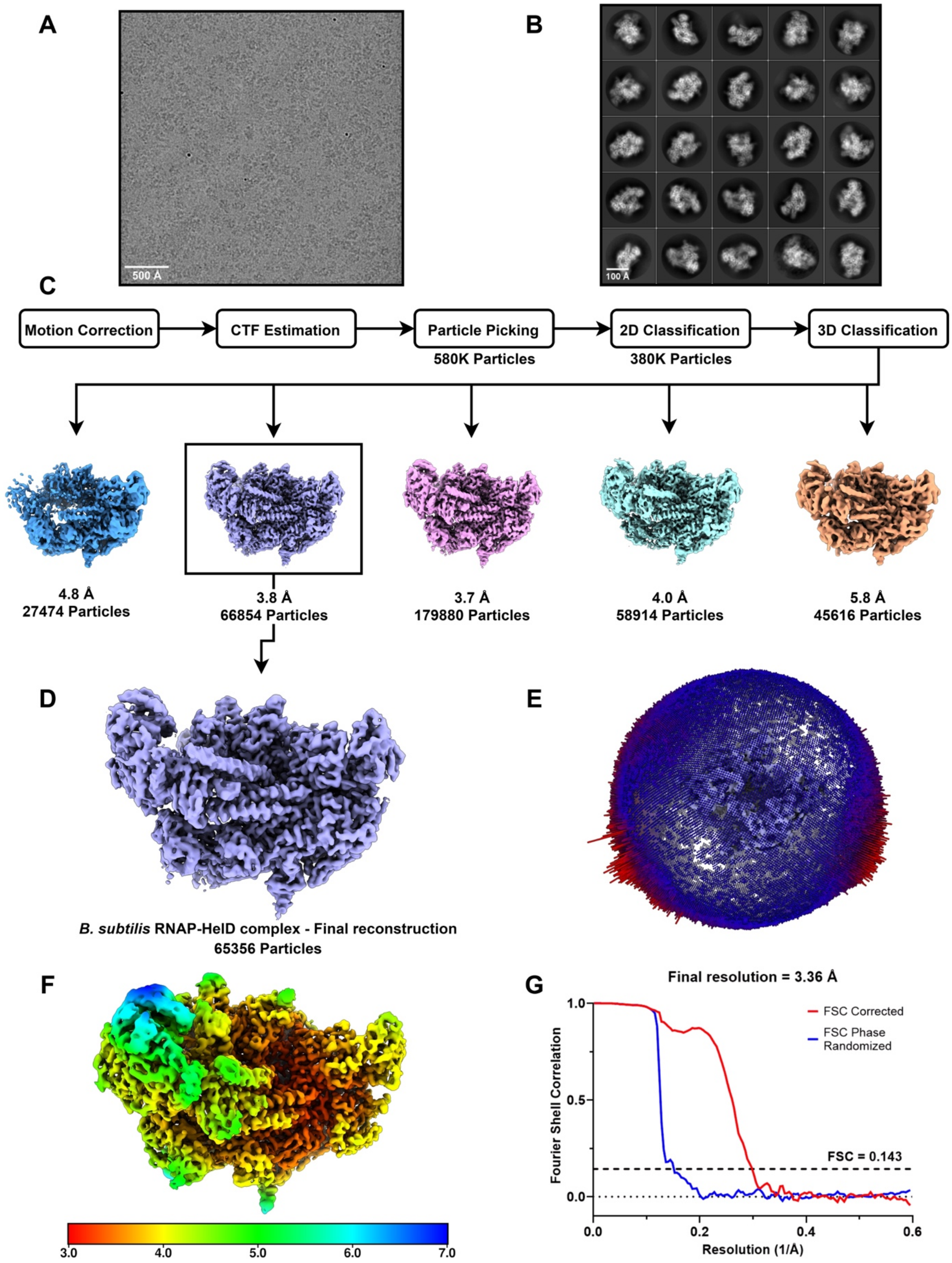

Supplementary Figure 3: Reconstruction of the *B. subtilis* RNAP-HelD complex.

a, Representative micrograph **b**, Representative class averages, ordered by particle number (descending, left to right, top to bottom). Scale bars (Å) are shown in the bottom left corner of

panels **a** and **b**. **c**, Simplified workflow for preprocessing and classification of the RNAP-HelD complex. **d**, Final refined, unsharpened map of the *B. subtilis* RNAP HelD complex. Final reconstruction was performed with a subset of 65,356 particles. **e**, Orientational distribution for the RNAP HelD complex. Relative particle frequency per orientation is shown by the height and color of the cylinder (higher frequency = longer cylinder, red, lower frequency = shorter cylinder, blue). **f**, Local resolution across the final RNAP HelD complex reconstruction. Local resolution estimation was performed in RELION. **g,** Fourier shell correlation curves for the RNAP HelD complex reconstruction. Resolution estimation was performed in RELION using the gold-standard fourier-shell correlation criterion (GS-FSC).

Supplementary Figure 4.

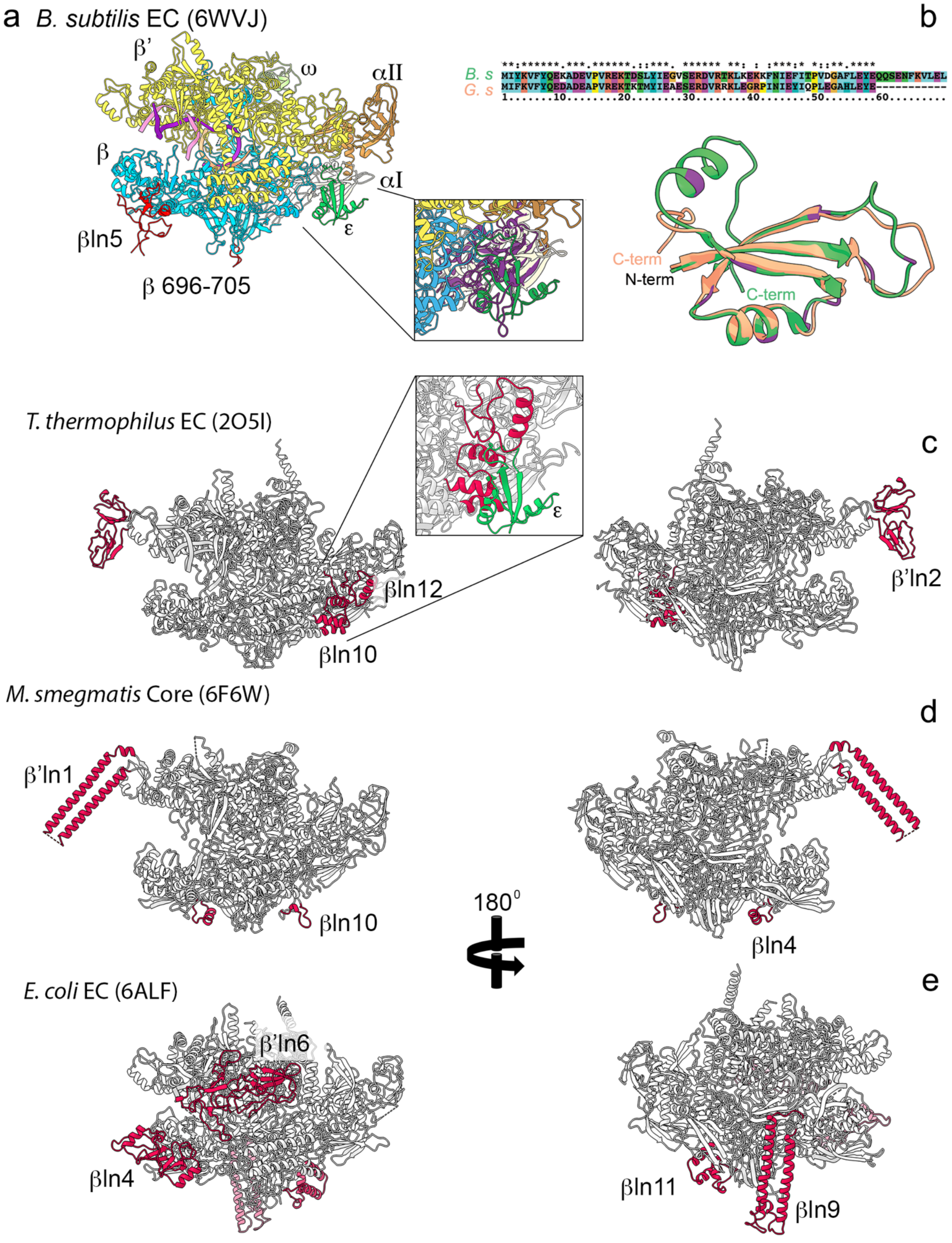

Supplementary Figure 4. Comparison of RNAP dimensions and ε/archaeal Rpo3 location. a, *B. subtilis* EC with ε docked based on similarity with the RNAP-HelD complex. αI (pale yellow),

αII (brown), β (azure), β' (yellow), ω (pale green), ε (green), template DNA (purple), non-template DNA (pink), RNA (orange). The location of the βln5 and β E696-G705 insertions are shown in red. In the insert the superimposed Rpo3 subunit of *Saccharolobus shibatae* RNAP (PDBB ID: 2WAQ) is shown in purple. Although there is no structural similarity, the location of ε closely matches that of the C-terminal half of archaeal RNAP Rpo3 (Y167-V222, PDBID: 2WAQ), and eukaryotic RNAP polII RPB3. RPB3 along with RPB10, -11, and -12 are required for assembly and stability of eukaryotic RNAP polII. **b,** shows the sequence and structure alignment of ε from *B. subtilis* (*B. s*) and *G. stearothermophilus* (*G. s*). *G.s* ε (PDB ID: 4NJC) is shown in orange, and *B. subtilis* ε from this work in green. The proteins are 67% identical (with 81% similarity) over the 58 amino acids for which their structures were determined. Locations of amino acid differences (neither identical nor similar) are shown in purple on the *B. subtilis* structure. All of the amino acids that were neither identical nor similar, other than *B. subtilis* T49 (*G. stearothermophilus* Q49), were solvent-exposed and are not involved in interaction with RNAP. **c-e,** *T. thermophilus* EC (PDB ID 2O5I), *M. smegmatis* core (PDB ID 6F6W), and *E. coli* EC (PDB ID 6ALF) with lineage-specific inserts shown in red. The insert in (**c**) shows ε superimposed on the structure illustrating the overlap with the postions of the βln10 and βln12 inserts.

Supplementary Figure 5.

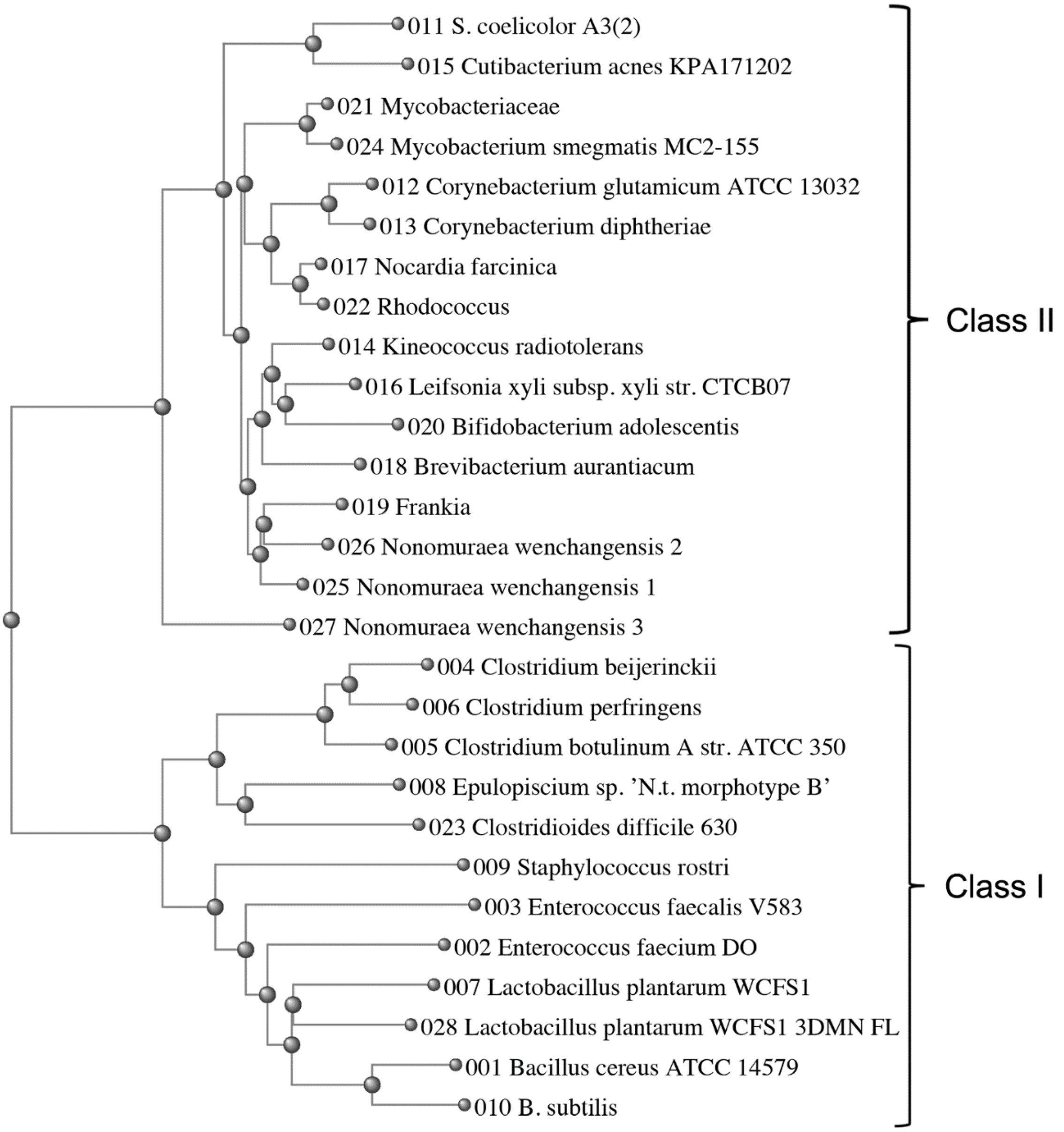

Supplementary Figure 5. Phylogenetic tree view (Grishin general) of selected HelD sequences. The HelD family of proteins is present predominantly in the Gram-positive eubacteria and is represented by 24436 entries with a sequence length of ~750 aa that segregates into at least two related classes (Class I and II, respectively). There are two additional minor families comprising 74 and 37 entries, respectively, with DUF4968 or DUF4968 + GH31_N superfamily motifs present as a C-terminal extension. These proteins are >1000 aa in length with a ~750 aa N-terminal region that is similar to HelD Class II proteins. These minor families are restricted exclusively to Gram positive obligate anerobes related to the *Clostridiales/Lachnospiraceae* and are important in general gut health. This smaller group of larger proteins are essentially indistinguishable from other HelD

(class II) proteins other than the fact that they have C-terminal extensions. Sequence numbers correspond to: 001, *Bacillus cereus* ATCC14579; 002, *Enterococcus faecium* DO; 003, *Enterococcus faecalis* V583; 004, *Clostridium beijerinckii*, 005, *Clostridium botulinum* A str. ATCC 3502; 006, *Clostridium perfringens*, 007; *Lactobacillus plantarum* WCFS1 *helD1*; 008 *Epulopiscium sp.* 'N.t. morphotype B'; 009, *Staphylococcus rostri*; 010, *Bacillus subtilis* 168; 011, *Streptomyces coelicolor* A3(2); 012, *Corynebacterium glutamicum* ATCC 13032; 013, *Corynebacterium diphtheriae*; 014, *Kineococcus radiotolerans*; 015, *Cutibacterium acnes* KPA171202; 016, *Leifsonia xyli subsp. xyli* str. CTCB07; 017, *Nocardia farcinica*; 018, *Brevibacterium aurantiacum*; 019, *Frankia sp.*; 020, *Bifidobacterium adolescentis*; 021, *Mycobacteriaceae*; 022, *Rhodococcus sp.*; *Clostridioides difficile 630*; 024, *Mycobacterium smegmatis MC2-155*; 025 *Nonomuraea wenchangensis helD1*; 026, *Nonomuraea wenchangensis helD2*; 027, *Nonomuraea wenchangensis helD3*; 028, *Lactobacillus plantarum WCFS1 helD2* (sequence from which PDB ID: 3DMN was derived). Sequence alignments are shown in Supplementary Figure 7.

Supplementary Figure 6.

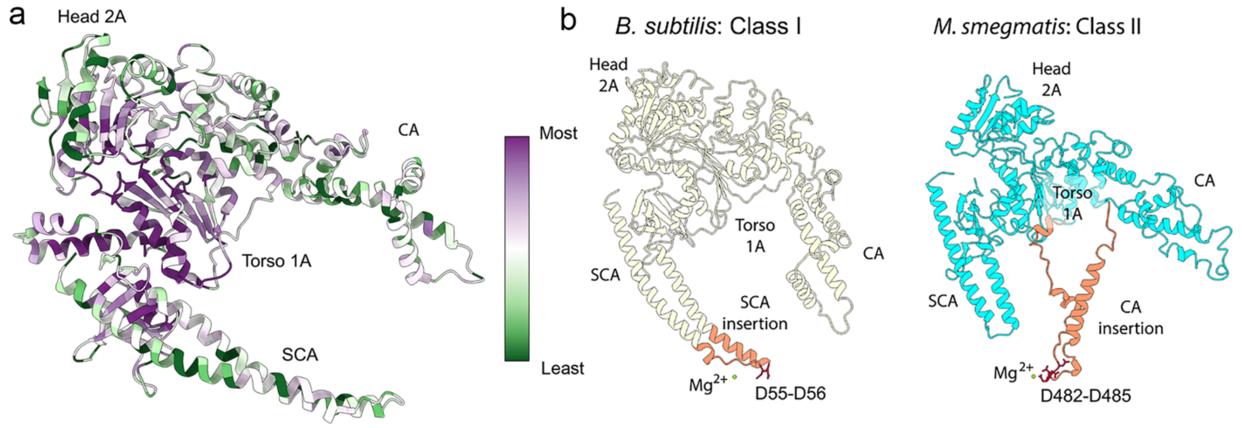

Supplementary Figure 6. HelD sequence conservation relative to structure and structural comparison of Class I and II HelD proteins. a, The image was constructed using the HelD chain from the RNAP-HelD complex with the alignment file from Supplementary Figure 7 in ConSurf. Most highly conserved amino acids relative to location, purple, and least conserved in green. **b,** The architecture of Class I and II HelD proteins has been addressed in the main text of this article (Class I) and in the accompanying paper by Kouba *et al* (Class II). The key structural differences that separate Class I and II HelD proteins involve the structural motif that causes nucleic acid displacement from the active site of RNAP are shown in orange close to the active site Mg^{2+} (small green sphere) with acidic residues labelled and shown as red sticks.

Supplementary Figure 7.

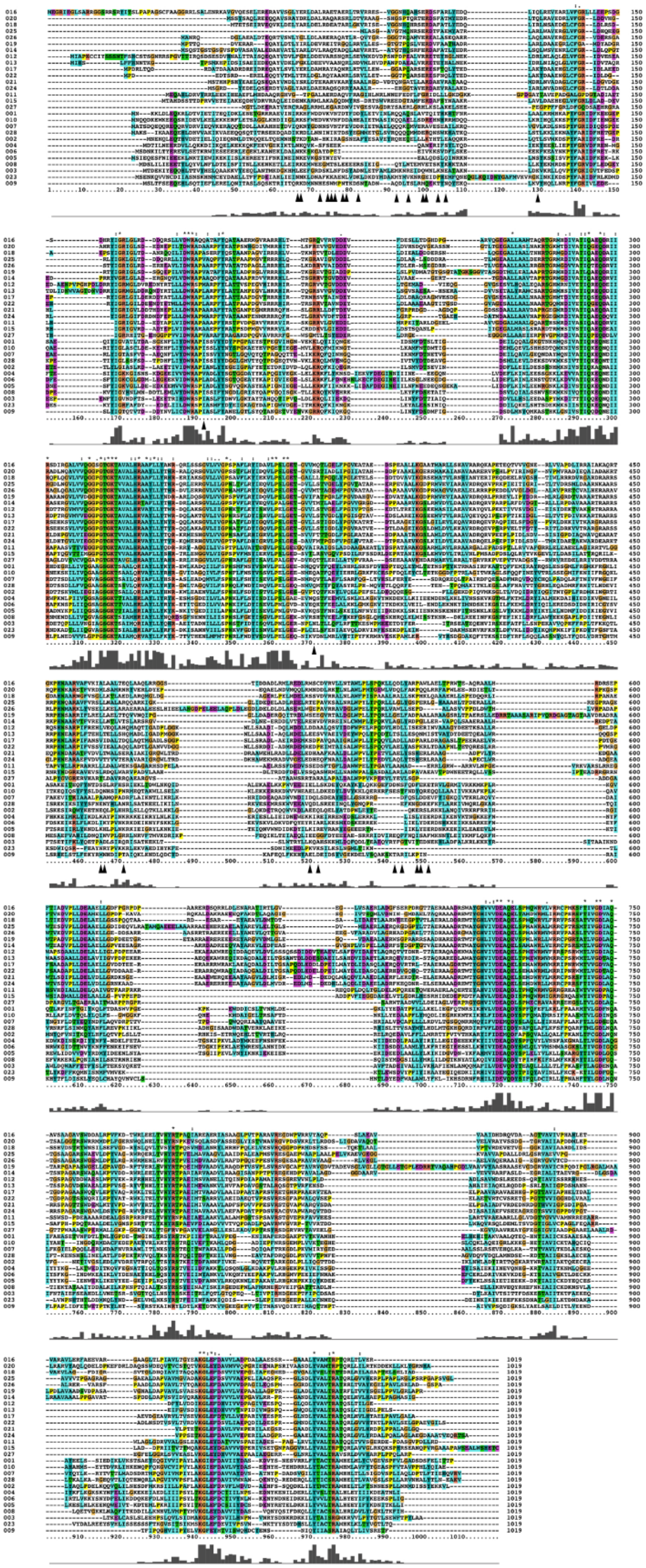

Supplementary Figure 7. Multiple alignment of HelD sequences. Sequence alignment was performed in clustalX. The histogram below the alignment illustrates the level of position-specific sequence conservation. Black arrowheads indicate the residues in *B. subtilis* HelD (sequence 010) involved in salt-bridge/hydrogen bond interactions with RNAP. Sequence numbers on the left hand side of the figure correspond to the following organisms: 001, *Bacillus cereus* ATCC14579; 002, *Enterococcus faecium* DO; 003, *Enterococcus faecalis* V583; 004, *Clostridium beijerinckii*, 005, *Clostridium botulinum* A str. ATCC 3502; 006, *Clostridium perfringens*, 007; *Lactobacillus plantarum* WCFS1 *helD1*; 008 *Epulopiscium sp.* 'N.t. morphotype B'; 009, *Staphylococcus rostri*; 010, *Bacillus subtilis* 168; 011, *Streptomyces coelicolor* A3(2); 012, *Corynebacterium glutamicum* ATCC 13032; 013, *Corynebacterium diphtheriae*; 014, *Kineococcus radiotolerans*; 015, *Cutibacterium acnes* KPA171202; 016, *Leifsonia xyli subsp. xyli* str. CTCB07; 017, *Nocardia farcinica*; 018, *Brevibacterium aurantiacum*; 019, *Frankia sp.*; 020, *Bifidobacterium adolescentis*; 021, *Mycobacteriaceae*; 022, *Rhodococcus sp.*; *Clostridioides difficile 630*; 024, *Mycobacterium smegmatis MC2-155*; 025 *Nonomuraea wenchangensis helD1*; 026, *Nonomuraea wenchangensis helD2*; 027, *Nonomuraea wenchangensis helD3*; 028, *Lactobacillus plantarum WCFS1 helD2* (sequence from which PDB ID: 3DMN was derived). Numbers below and on the right indicate the relative sequence length of the multiple sequence alignment.

Supplementary Figure 8.

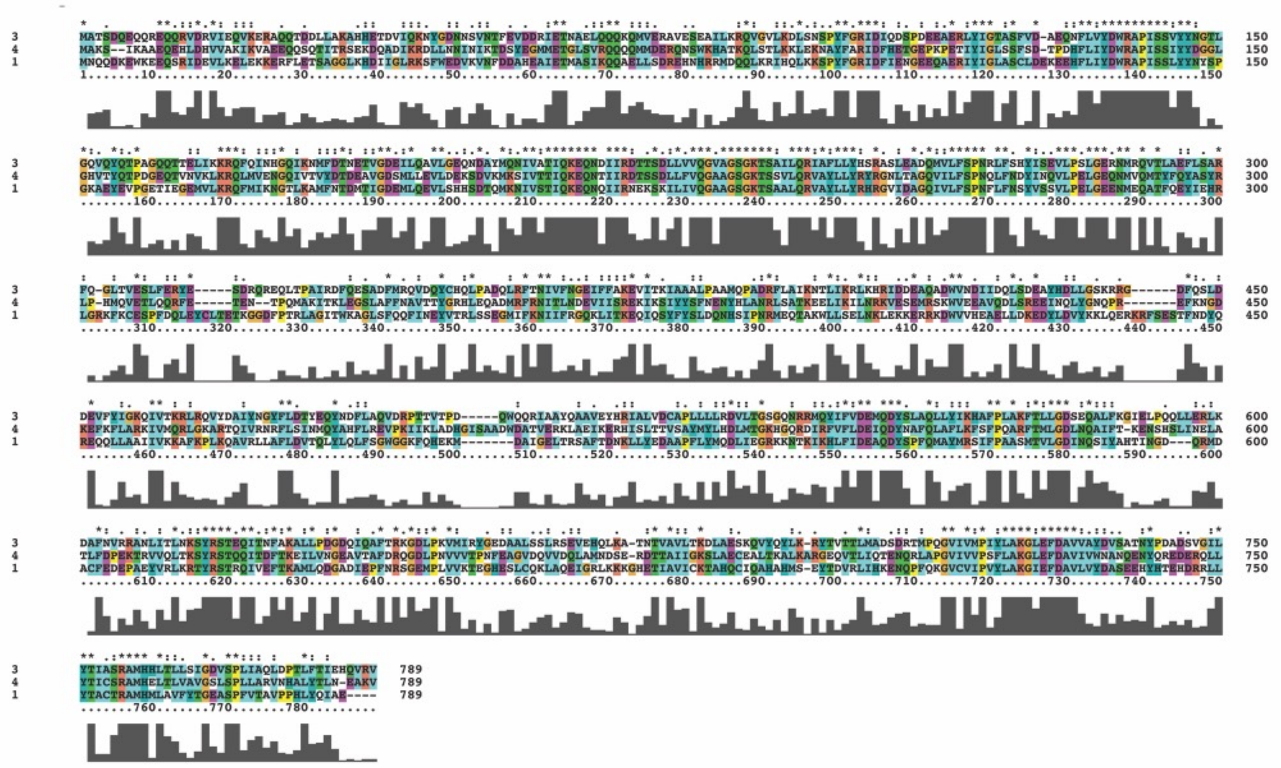

Supplementary Figure 8. Sequence alignment of *B. subtilis* and *L. plantarum* HelDs. In some organisms HelD is encoded by more than one gene (*e.g. Lactobacillus plantarum* Class I, and *Nonomuraea wenchangensis* Class II; Extended Data Fig. 6). The two copies of the *helD* gene in *L. plantarum*, lp_0432 and lp_0910 are are ~0.5 Mb apart (locations at 384 kb and 838 kb, respectively) and are surrounded by different genes on their up- and downstream sides. Sequence alignment of these two HelD sequences with *B. subtilis* HelD shows that even in these closely related organisms, for which the structure of the C-terminal domain of lp_0910 (PDB ID: 3DMN) was used to help refine the C-terminal domain of *B. subtilis* HelD, there is remarkably low sequence conservation, especially within the region comprising the CA domain (~aa300-500). Sequence alignment was performed in clustalX. Sequence numbers correspond to the following organisms: 001, *Bacillus subtilis* 168; 003, *Lactobacillus plantarum WCFS1 helD1*; 004, *Lactobacillus plantarum WCFS1 helD2*. Relative sequence conservation is shown as a histogram below the alignments, and the numbers below and on the right indicate the relative sequence length of the multiple sequence alignment.

Supplementary Table 2. Conserved HelD sequence motifs. Despite the relatively low level of overall similarity between HelD sequences from even closely related organisms, a series of motifs could be identified that define HelD proteins and also enable classification into one of two classes (Classes I and II). Sequence numbers refer to the *B. subtilis* HelD sequence (010 in multiple alignment Supplementary Figure 7). X corresponds to a poorly conserved sequence (any amino acid) and h to a conserved hydrophobic residue. Residues coloured red are specific to class I and green to class II sequences.

Motif	Position (*B. subtilis* numbering)	Sequence	
I	098-102	${}^{P}_{X}\;{}^{Y}_{X}\;F\;{}^{G}_{A}\;{}^{R}_{K}$	
II	118-121	${}^{Y}_{H}\;{}^{I}_{h}\;G\;{}^{R}_{X}$	
III	135-146	$h\;X\;D\;W\;R\;{}^{A}_{S}\;{}^{P}_{X}\;X\;{}^{A}_{S}\;X\;X\;{}^{F}_{Y}\;Y$	
IV	209-222	${}^{I}_{V}\;{}^{V}_{I}\;X\;T\;{}^{I}_{L}\;Q\;X\;E\;Q\;{}^{D}_{N}\;X\;{}^{I}_{V}\;{}^{I}_{V}\;R$	
V	233-240	$G\;X\;{}^{P}_{A}\;G\;{}^{T}_{S}\;G\;K\;T$	Walker A site
VI	244-255	${}^{L}_{M}\;{}^{H}_{Q}\;R\;X\;A\;{}^{Y}_{F}\;L\;L\;{}^{Y}_{F}\;X\;X\;{}^{R}_{K}$	
VII	279-285	${}^{V}_{I}\;L\;P\;X\;L\;G\;{}^{E}_{X}$	
VIII	543-550	$h\;h\;{}^{V}_{I}\;D\;E\;{}^{A}_{h}\;Q\;{}^{D}_{E}$	Walker B site
IX	568-576	$T\;X\;X\;G\;D\;X\;{}^{A}_{x}\;Q$	
X	603-610	$L\;X\;X\;X\;{}^{Y}_{F}\;R\;{}^{T}_{S}\;{}^{P}_{X}$	
XI	713-718	$K\;G\;{}^{L}_{h}\;E\;{}^{F}_{Y}\;D$	
XII	740-747	$Y\;{}^{V}_{X}\;X\;X\;{}^{T}_{S}\;R\;{}^{A}_{X}\;{}^{T}_{h}$	

Supplementary Table 3. Solvent accessible surface area (SASA; in Å^2) for each protein, buried by protein-protein interactions. **a**, the elongation complex and **b**, the RNAP-HelD complex. For the subunits in each row, the buried SASA is given and the column heading gives the interaction partner responsible for the buried surface and the column heading gives the interaction partner responsible for the buried surface.

**a**							
		ω		β'	β	α2	α1
α1					1502	1614	-
α2				1055	232	-	1609
β		199		7721	-	202	1408
β'		1551		-	7505	1038	
ω		-		1521	189		
**b**							
	HelD	ω	ε	β'	β	α2	α1
α1			664	67	1504	1596	-
α2			24	1064	211	-	1553
β	685	199	546	7274	-	202	1422
β'	3576	1523	134	-	7018	1060	58
ε			-	144	588	24	702
ω		-		1623	206		
HelD	-			3711	717		

Supplementary Figure 9.

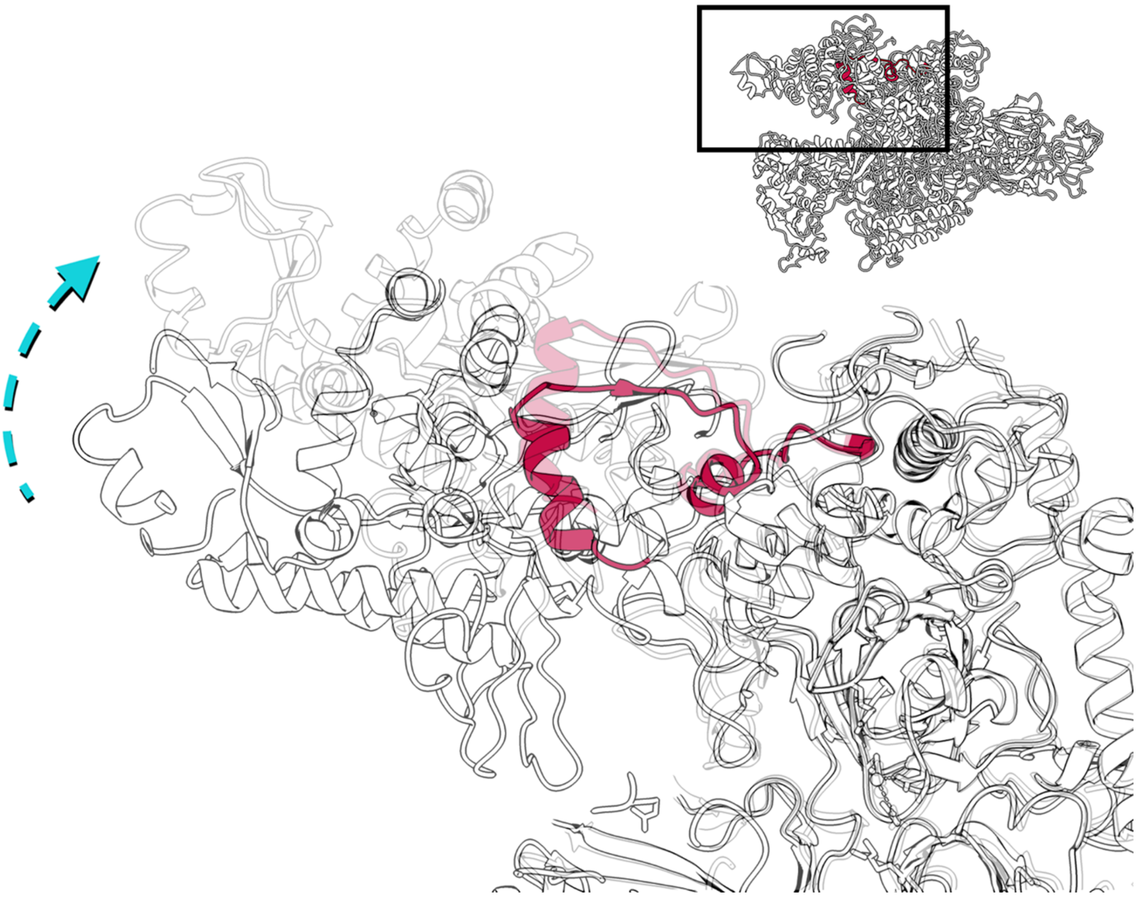

Supplementary Figure 9. HelD-induced clamp movement is due to SW5 movement. The conserved SW5 motif is located in the C-terminal portion of the β’ subunit from Q1140-T1175 (red) and forms a hinge upon which the β’ clamp is able to swing up and down. Superimposing the SW5- β’ clamp regions of the EC and RNAP-HelD structures shows the hinge movement that enables the dramatic opening of the β’ clamp upon HelD binding and CA movement, and that conformational changes are largely restricted to the β’ clamp domain (boxed region). The β clamp from the elongation complex is shown overlayed with the same region of the RNAP-HelD complex as a ghost image. Clamp movement is illustrated by the dashed cyan arrow. The inset shows the elongation complex (with nucleic acids removed for clarity) with the portion of RNAP shown in the main panel boxed.

Supplementary Figure 10.

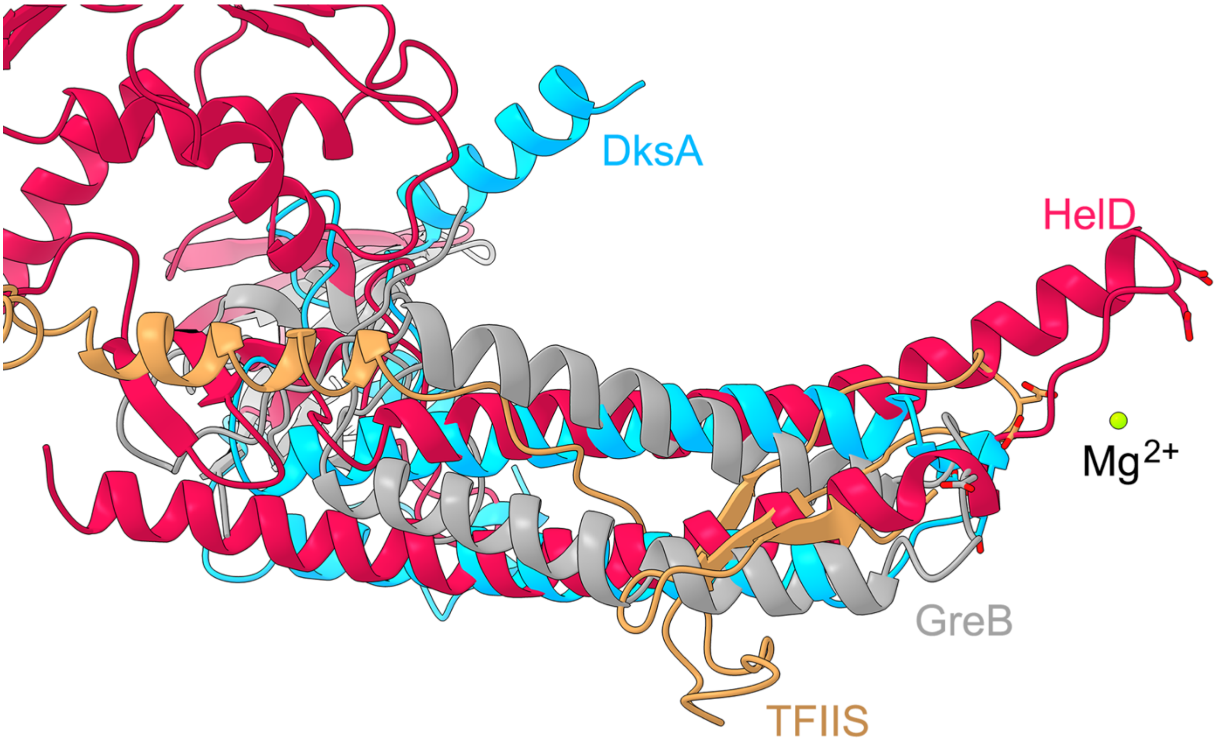

Supplementary Figure 10. Compilation of structural overlays of secondary channel binding domains. All overlays were produced from proteins in complex with their cognate RNAP and are presented with respect to the RNAP-HelD complex. HelD is shown in red and the active site Mg^{2+} in green. GreB (PDB ID 6RI7) is shown in grey, DksA (PDB ID 5W1T) in blue, and TFIIS (PDB ID 1Y1V) in brown. Acidic residues located at the tips of the respective secondary channel arms are shown as sticks.

Supplementary Table 4. HelD atoms (left column) forming hydrogen bonds and salt bridges with residues in β and β' (right column). SCA interactions in dark grey, with residues that surround the active site β' 447-NADGDFD-453 and cage the catalytic Mg^{2+} in black. Interactions with CA residues highlighted in grey, and other regions, no colour. Distance (Å), centre column.

HelD	Distance Å	β or β'	
R44Nη1	3.44	β’M1057O	SCA
K45Nζ	3.48	β’E1061Oε2	
K45Nζ	3.68	β’G1059O	
K52Nζ	3.81	β’βC443O	
R78Nη1	3.17	β’D710Oδ1	
R78Nη1	3.9	β’Q713Oε1	
N81Nδ2	3.68	β’D710Oδ2	
N81Nδ2	3.56	β’D710Oδ1	
R83Nη1	3.65	β’G942O	
R91Nη1	3.38	β’E699Oε2	
R91Nη2	3.34	β’E699Oε2	
D50O	3.89	β’R735Nη2	
V53O	2.78	β’R414Nη2	
N54Oδ1	2.95	β’R414Nη1	
D56Oδ1	3.7	β’R341Nη1	
D56Oδ1	3.54	β’R341Nη2	
D56Oδ2	3.56	β’Q454Nε2	
E73Oε2	3.22	β’R748Nη2	
D77Oδ2	3.56	β’K709Nζ	
R78Nη2	3.91	β’D710Oδ1	
R91Nη2	3.34	β’E699Oε2	
Q70Oε1	3.46	βR636Nε	
K52Nζ	3.1	βE772Oε2	
D57Oδ2	3.67	βK1047Nζ	
E60Oε2	3.41	βK924Nζ	
Q70Nε2	3.87	βR965O	
E60Oε2	3.41	βK924Nζ	
Y449Oη	2.62	β’G109O	CA
Q450Nε2	3.71	β’G299O	
E421Oε2	3.54	β’R205Nη1	
L423O	3.88	β’K208Nζ	
T445O	3.12	β’K321Nζ	
E443Oε1	3.6	β’K314Nζ	
E452Oε1	2.99	β’R1144Nη2	
E452Oε1	2.99	β’R1144Nη2	
I141N	3.4	β’G690O	Other
Q290Nε2	3.87	β’V984O	
Q290O	3.47	β’R985Nη2	
Q290Oε1	2.42	β’R985Nη2	
N385Nδ2	2.46	βE227Oε2	
H386Nε2	3.14	βN225O	
N390Nδ2	3.71	βD222O	

Supplementary Table 5. HelD-RNAP hydrophobic interactions. RNAP and HelD chains are labelled β, β’ and H, respectively.

Atom 1	Atom 2
H GLN 70 NE2	β ASP 581 OD2
H GLU 443 O	β’ LYS 311 NZ
H GLU 421 OE1	β’ ARG 202 NH1
β’ LYS 318 NZ	H ASP 448 OD2
H LEU 422 CD1	β’ ARG 202 N
H LEU 422 CG	β’ THR 201 CG2
β’ ARG 1141 NH1	H TYR 428 CG
H TYR 428 CB	β’ ARG 1141 NH1
β’ LEU 310 O	H PHE 446 CE2
β’ PRO 108 CG	H GLU 421 OE2
β’ ARG 1141 NH1	H TYR 428 CD2
H GLU 443 O	β’ LYS 311 CE
H TYR 428 CB	β’ ARG 1141 NH2
β’ LYS 105 CG	H TYR 449 CE2
H LEU 422 CD1	β’ THR 201 CB
H TYR 432 CE1	β’ GLU 1138 CB
H GLU 421 OE2	β’ PRO 119 CG
H LEU 422 CD1	β’ ARG 202 CB
β’ SER 118 CB	H HIS 418 CE1
H GLU 443 CG	β’ HIS 315 O
β’ PRO 108 CD	H GLU 421 CB
H LEU 422 CD1	β’ ARG 202 CA
β’ PRO 119 CB	H VAL 417 CG1
β’ ILE 107 CG1	H TYR 428 CE1
β’ LYS 318 NZ	H ASP 448 CG
H GLU 419 CG	β’ GLN 198 OE1
H PHE 446 CD2	β’ HIS 315 CE1
β’ GLN 1137 CG	H LEU 429 CD1
β’ ILE 107 CG1	H TYR 428 OH
β’ THR 201 CB	H LEU 422 CG
H LEU 422 CD2	β’ GLN 198 OE1
H GLU 421 CD	β’ ARG 202 NH1
β’ PRO 108 CG	H GLU 421 CD
H LEU 422 CD2	β’ THR 201 CG2
H VAL 417 CG1	β’ PRO 119 CG
H LEU 422 CD1	β’ THR 201 CG2
β’ LYS 311 NZ	H GLU 443 CG
H TYR 428 CD1	β’ ARG 1141 NH2
β’ GLY 319 CA	H GLU 443 CB
H TYR 428 CD1	β’ ARG 1141 NH1
H LEU 422 CD2	β’ THR 201 CB

β’ LEU 1125 CD1	H LEU 429 CD2
β’ GLY 296 CA	H PHE 446 CB
H GLU 421 OE2	β’ PRO 119 CD
β’ LEU 113 CD2	H GLU 421 OE1
β’ ILE 107 CG1	H TYR 428 CZ
β’ GLY 106 CA	H TYR 449 CE1
H LEU 422 CD2	β’ GLN 198 NE2
H PHE 446 CE2	β’ LYS 311 CD
H GLU 421 CG	β’ PRO 108 CD
β’ PRO 309 CB	H PHE 446 CZ
H LEU 429 CD2	β’ LEU 1125 CD2
β’ GLN 198 CD	H LEU 422 CD2
β’ PRO 309 CB	H PHE 446 CE1
H GLU 443 CD	β’ GLY 319 CA
H PHE 446 CD2	β’ HIS 315 NE2
H PHE 446 CZ	β’ LYS 311 CE
β’ ASN 295 ND2	H PHE 446 CD2
β’ GLU 1138 CB	H TYR 432 CD1
H PHE 446 CE1	D PRO 309 CG
β’ THR 201 C	H LEU 422 CD1
β’ LYS 311 NZ	H GLU 443 C
H TYR 428 CG	β’ ARG 1141 NH2
β’ ARG 300 NH2	H GLN 450 CG
β’ ARG 1141 CZ	H TYR 428 CB
H GLU 443 CG	β’ GLY 319 CA
β’ GLU 1138 CA	H TYR 432 CE1
H TYR 449 CE2	β’ LYS 105 CD
β’ GLN 198 CD	H GLU 419 CG
H GLU 443 CG	β’ HIS 315 CB
β’ LYS 311 CD	H GLU 443 O
β’ ARG 300 NH2	H GLN 450 CD
H GLU 419 CG	β’ GLN 198 NE2
H PHE 446 CE1	β’ PRO 309 CD
β’ PRO 108 CG	H GLU 421 CG
β’ LYS 105 CG	H TYR 449 CZ
β’ GLN 1137 CG	H LYS 425 CE
β’ ARG 1141 CZ	H TYR 428 CD1
H HIS 418 CE1	β’ SER 118 OG
H PHE 446 CZ	β’ LYS 311 CD
β’ LYS 311 CE	H SER 444 OG
β’ LYS 311 NZ	H GLU 443 CD
β’ ARG 1141 NH1	H TYR 428 CE2
H TYR 432 CD1	β’ GLU 1138 OE1
H TYR 432 CD1	β’ GLU 1138 CD
H GLU 421 OE2	β’ PRO 108 CD
β’ PRO 309 CG	H PHE 446 CD1
β’ ILE 107 CD1	H LEU 456 CD1
H GLU 421 CD	β’ PRO 108 CD

Supplementary Figure 11.

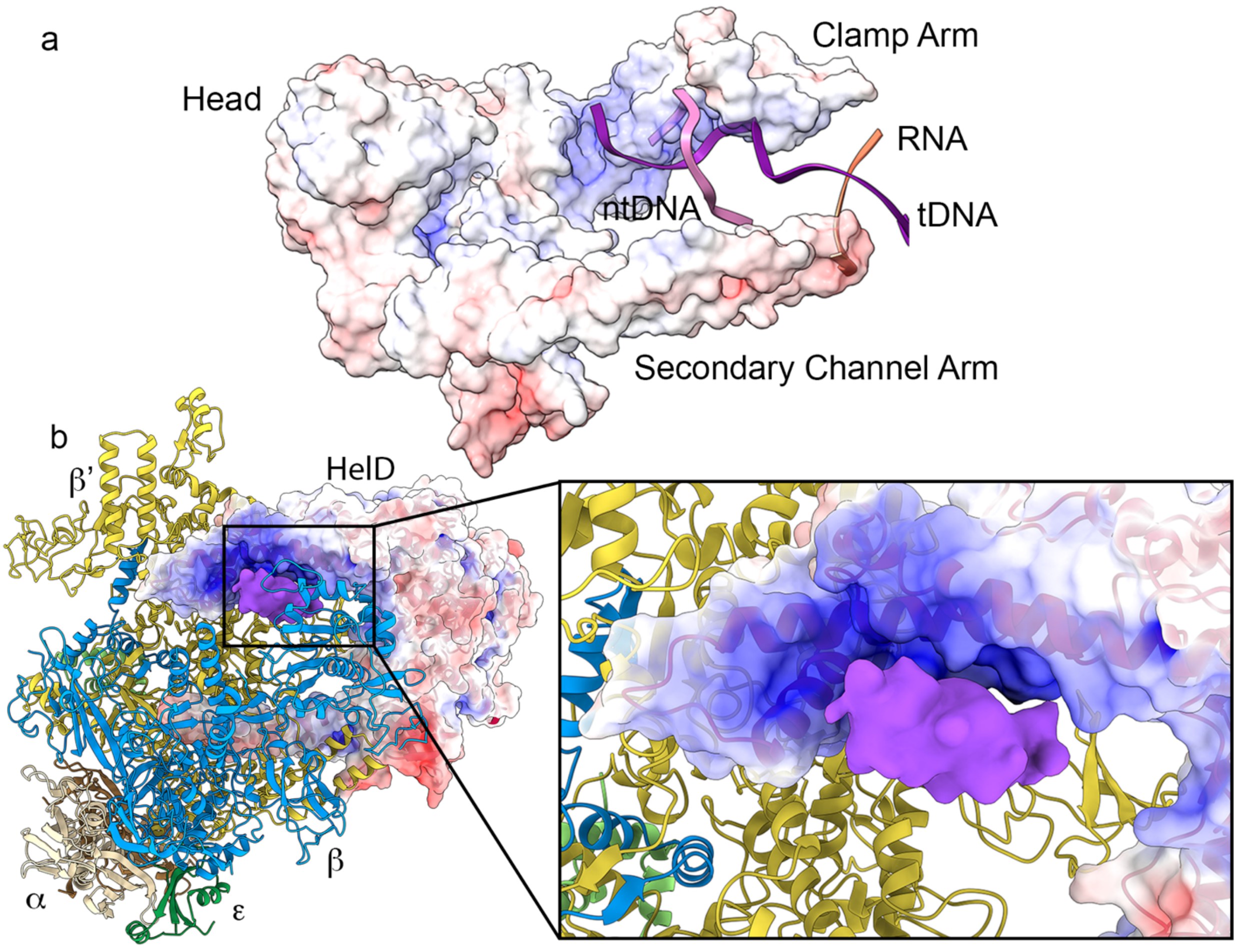

Supplementary Figure 11. Visible orphan density in the RNAP-HelD complex. a, ABPS surface electrostatic charge of HelD from red, most negative, to blue, most positive surface charge is shown and was generated using APBS-PDB2PQR (server.poissonboltzmann.org). The nucleic acids from the RNAP EC are also shown superimposed to illustrate the close juxtaposition between the positively charged region of the Clamp Arm with DNA. **b,** At low thresholds, orphan density was visible adjacent to the Clamp Arm in the unsharpened, unmasked electron density map. The orphan density is shown in purple, and all other subunits of RNAP coloured as in previous figures.

Supplementary Figure 12.

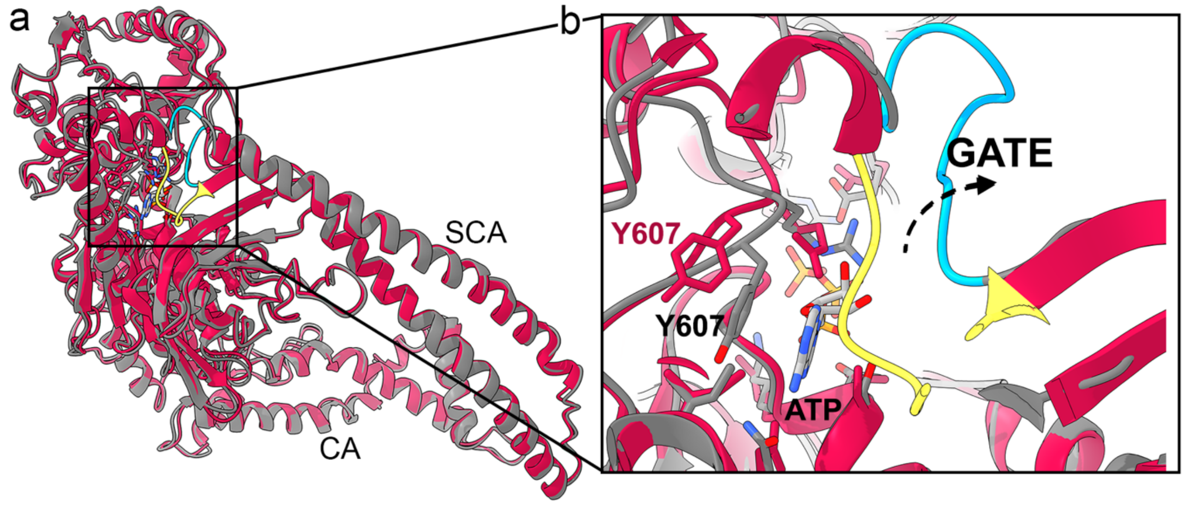

Supplementary Figure 12. ATP binding to HelD. a, HelD (grey) and PcrA (not shown) with ATP bound (PDB ID 1QHH) were superimposed to place ATP in the binding site, followed by energy minimisation and manual displacement of HelD ATP gate residues F183-G190 (blue). Unmodified HelD (red) is also shown with the ATP gate (yellow) in the closed position. **b,** Expanded view of the area boxed in **a** showing ATP with Y607 which stacks with the purine ring on binding. Dashed arrow indicates the movement of the gate residues from closed to open.
