# Supplementary material for: Molecular basis for RNA polymerase-dependent transcription complex recycling by the helicase-like motor protein HelD
Source: Nat Commun. 2020 Dec 18;11:6420. doi: 10.1038/s41467-020-20157-5 (PMC7749167; doi:10.1038/s41467-020-20157-5)
Supplement: Supplementary file 3 — Description of Additional Supplementary Files [file 41467_2020_20157_MOESM3_ESM.pdf]

Description of Additional Supplementary Files

Supplementary Movie 1: RNAP elongation complex. The movie shows a 360° rotation of the EC.

Supplementary Movie 2: RNAP-HelD complex. The movies shows a 360° rotation of the RNAP-HelD complex, and highlights the Clamp and Secondary Channel arms of HelD.

Supplementary Movie 3: RNAP remodelling by HelD. The movie shows the conformational changes induced in RNAP by HelD.

Supplementary Movie 4: 3D variability analysis of the RNAP-HelD complex. The movie shows the results of 3D variability analysis of the RNAP-HelD complex which highlights how secondary channel arm anchoring provides a platform for primary channel opening by movement of the clamp arm.
