# Supplementary material for: Molecular basis for RNA polymerase-dependent transcription complex recycling by the helicase-like motor protein HelD
Source: Nat Commun. 2020 Dec 18;11:6420. doi: 10.1038/s41467-020-20157-5 (PMC7749167; doi:10.1038/s41467-020-20157-5)
Supplement: Supplementary file 8 — Reporting summary [file 41467_2020_20157_MOESM8_ESM.pdf]

nature research

Corresponding author(s): Peter Lewis and Gokhan Tolun

Last updated by author(s): Oct 26, 2020

Reporting Summary

Nature Research wishes to improve the reproducibility of the work that we publish. This form provides structure for consistency and transparency in reporting. For further information on Nature Research policies, see Authors & Referees and the Editorial Policy Checklist .

Statistics

For all statistical analyses, confirm that the following items are present in the figure legend, table legend, main text, or Methods section.

n/a	Confirmed	
☐	☒	The exact sample size (*n*) for each experimental group/condition, given as a discrete number and unit of measurement
☐	☒	A statement on whether measurements were taken from distinct samples or whether the same sample was measured repeatedly
☒	☐	The statistical test(s) used AND whether they are one- or two-sided<br>*Only common tests should be described solely by name; describe more complex techniques in the Methods section.*
☒	☐	A description of all covariates tested
☒	☐	A description of any assumptions or corrections, such as tests of normality and adjustment for multiple comparisons
☐	☒	A full description of the statistical parameters including central tendency (e.g. means) or other basic estimates (e.g. regression coefficient) AND variation (e.g. standard deviation) or associated estimates of uncertainty (e.g. confidence intervals)
☒	☐	For null hypothesis testing, the test statistic (e.g. *F*, *t*, *r*) with confidence intervals, effect sizes, degrees of freedom and *P* value noted<br>*Give P values as exact values whenever suitable.*
☒	☐	For Bayesian analysis, information on the choice of priors and Markov chain Monte Carlo settings
☒	☐	For hierarchical and complex designs, identification of the appropriate level for tests and full reporting of outcomes
☒	☐	Estimates of effect sizes (e.g. Cohen's *d*, Pearson's *r*), indicating how they were calculated

Our web collection on statistics for biologists contains articles on many of the points above.

Software and code

Policy information about availability of computer code

Data collection

EPU for Life Sciences v2.4.0 (Thermo Fisher)
NCBI CDART (https://www.ncbi.nlm.nih.gov/Structure/lexington/lexington.cgi)

Data analysis

MotionCor2 v2.1.10
GCTF v1.06
CrYOLO 1.6.1
RELION v3.1
Cryosparc v2.14
PHENIX v1.18
Coot v0.8.9
ISOLDE v1.0b4
Chimera v1.13.1
ChimeraX v0.93
NAMD v2.13
PISA (https://www.ebi.ac.uk/pdbe/pisa/)
APBS-PDB2PQR (http://server.poissonboltzmann.org)
ClustalX v2.1
NCBI COBALT (https://www.ncbi.nlm.nih.gov/tools/cobalt/re_cobalt.cgi)
ConSurf (https://consurf.tau.ac.il)

For manuscripts utilizing custom algorithms or software that are central to the research but not yet described in published literature, software must be made available to editors/reviewers. We strongly encourage code deposition in a community repository (e.g. GitHub). See the Nature Research guidelines for submitting code & software for further information.

Data

Policy information about availability of data

All manuscripts must include a data availability statement. This statement should provide the following information, where applicable:

- Accession codes, unique identifiers, or web links for publicly available datasets
- A list of figures that have associated raw data
- A description of any restrictions on data availability

CryoEM maps were deposited in the Electron Microscopy Data Bank (https://www.ebi.ac.uk/pdbe/emdb/) under accession codes EMD-21921 (RNAP-HelD) and EMD-21920 (RNAP elongation complex), and will be released upon publication. Structure coordinates have been deposited in the RCSB Protein Data Bank (https://www.rcsb.org/) with accession codes 6WVK (RNAP-HelD) and 6WVJ (dimeric RNAP elongation complex), and will be released upon publication. Bacillus subtilis HelD protein sequences (O32215) was obtained from UniProtKB (https://www.uniprot.org/) and used to identify HelD-like proteins in NCBI CDART (https://www.ncbi.nlm.nih.gov/Structure/lexington/lexington.cgi?). Phylogenetic trees were constructed using NCBI COBALT (https://www.ncbi.nlm.nih.gov/tools/cobalt/re_cobalt.cgi).

Field-specific reporting

Please select the one below that is the best fit for your research. If you are not sure, read the appropriate sections before making your selection.

☒ Life sciences ☐ Behavioural & social sciences ☐ Ecological, evolutionary & environmental sciences

For a reference copy of the document with all sections, see nature.com/documents/nr-reporting-summary-flat.pdf

Life sciences study design

All studies must disclose on these points even when the disclosure is negative.

Sample size	Information on sample size is presented in Extended Data Figures 2 (RNAP-HelD complex) and 3 (RNAP elongation complex). No sample size calculations were performed. The number of images, and consequently, the number of particles initially collected (i.e., sample size) was based on the average number of particles visible in the first few micrographs obtained from different regions of the grid. Assuming a large proportion of the data will be unsuitable for processing due to bad particles (see below), a conservative estimate is made on the number of images that will be required for reconstruction to ~3.5A. The processing of this image data yielded anisotropy-free (or low-enough anisotropy) high-resolution structures that allowed atomic modelling, indicating that the image data size was sufficiently large, with adequate numbers of particles in random orientations.
Data exclusions	Structure determination by Cryo-EM routinely involves the sorting of high quality particles suitable for use in reconstructions from poor ones. Poor quality particles may be due to the inherent heterogeneity in the sample being used or caused by the process of grid preparation where particles at the air-water interface are damaged. The processing steps involving data exclusion are outlined in Extended Data Figures 2 and 3, and the number of particles eliminated at each step can be seen in these figures.
Replication	During cryo-EM image processing, data is randomly divided into two halves, and a structure is determined from each one. Then, these two structures are compared to each other for determination of resolution, which is a built-in assessment of reproducibility.
Randomization	The cryo-EM image data has been collected using automated software, after selecting areas in the cryo-EM grids that are thin-enough for imaging. Therefore, no user-bias was introduced during data collection since images of all of the suitable holes in the grids were automatically acquired. Moreover, reconstruction of anisotropy-free (or low-enough anisotropy) high-resolution structures that allowed atomic modeling from these particles indicates that the orientations of the particles were random-enough to allow for such an outcome.
Blinding	Not applicable for cryo-EM where samples have to be extensively screened to identify regions of vitrified ice of appropriate thickness with a suitable density of mono-disperse particles.<br><br>No blinding was used in the enzyme assays to determine ATPase and transcriptional activity. These are enzyme activity assays and blinding is not applicable. Appropriate controls were included to ensure the data presented represented actual enzyme activity as detailed in the methods and figure legends.

Reporting for specific materials, systems and methods

We require information from authors about some types of materials, experimental systems and methods used in many studies. Here, indicate whether each material, system or method listed is relevant to your study. If you are not sure if a list item applies to your research, read the appropriate section before selecting a response.

Materials & experimental systems

n/a	Involved in the study
☒	☐ Antibodies
☒	☐ Eukaryotic cell lines
☒	☐ Palaeontology
☒	☐ Animals and other organisms
☒	☐ Human research participants
☒	☐ Clinical data

Methods

n/a	Involved in the study
☒	☐ ChIP-seq
☒	☐ Flow cytometry
☒	☐ MRI-based neuroimaging
