# Supplementary material for: Molecular basis for RNA polymerase-dependent transcription complex recycling by the helicase-like motor protein HelD
Source: Nat Commun. 2020 Dec 18;11:6420. doi: 10.1038/s41467-020-20157-5 (PMC7749167; doi:10.1038/s41467-020-20157-5)

Uncropped Gels
Lanes used to construct Supplementary figure 1a marked by dashed boxes.

1. RNA polymerase elongation complex

EC

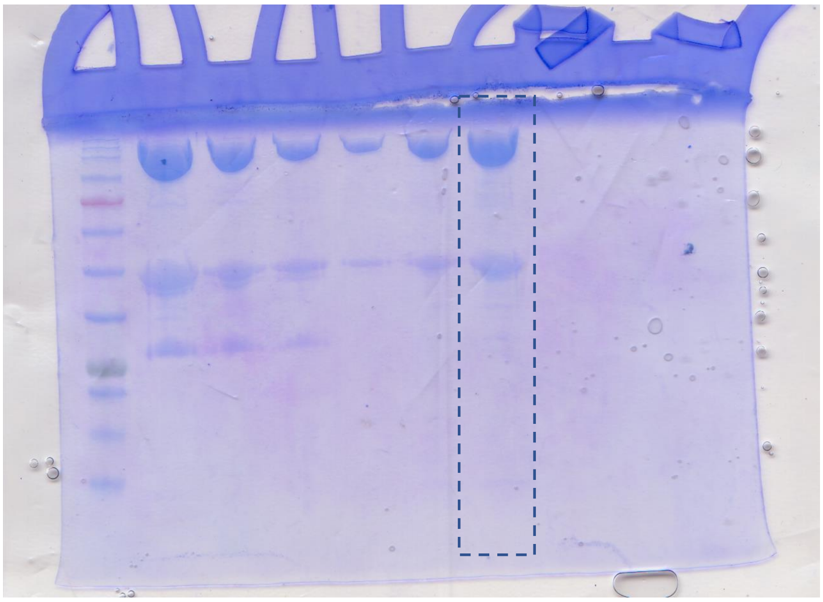

2. HE

HE

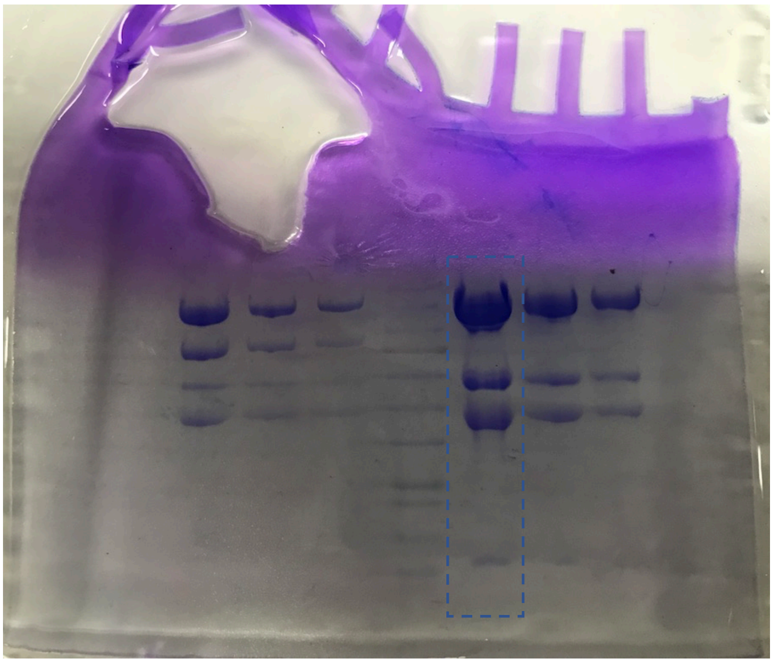

3. RNAP-HelD complex and HE

RNAP-HelD

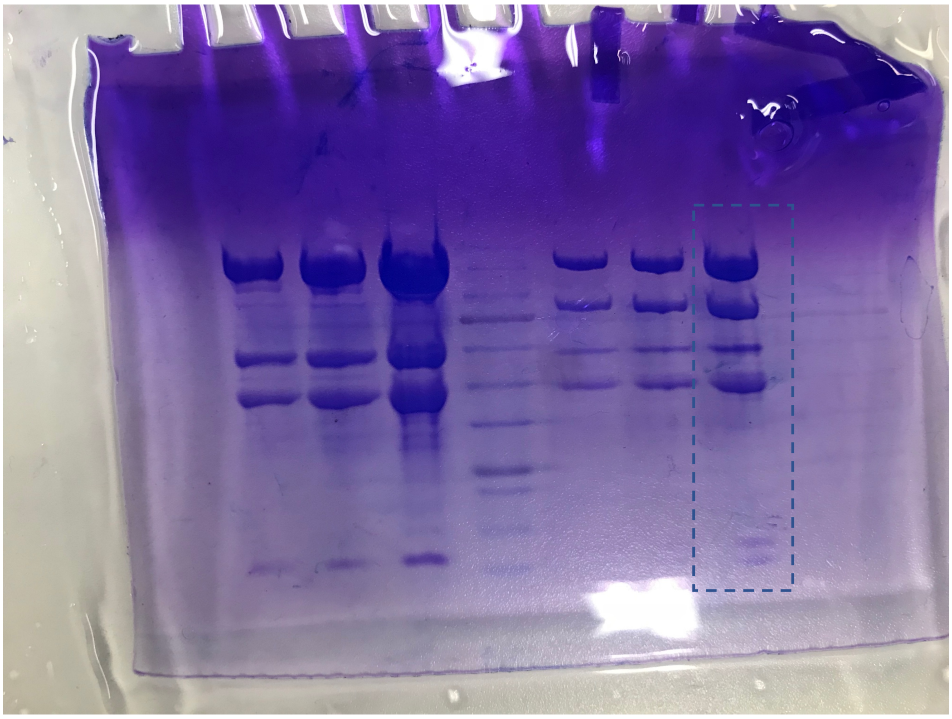

Supplement: Supplementary file 9 — Source Data [file 41467_2020_20157_MOESM9_ESM.zip › Raw Data/Uncropped Gels.pdf]
